# Microvascular anastomosis of the human lacrimal gland: a concept study towards transplantation of the human lacrimal gland

**DOI:** 10.1007/s00417-022-05933-x

**Published:** 2022-12-07

**Authors:** Christoph Holtmann, Mathias Roth, Timm Filler, Ann Kathrin Bergmann, Daniel Hänggi, Sajjad Muhammad, Maria Borrelli, Gerd Geerling

**Affiliations:** 1 Department of Ophthalmology, University Hospital, Heinrich-Heine-University Duesseldorf, Moorenstr. 5, 40225 Duesseldorf, Germany; 2 Institute of Anatomy I, Heinrich-Heine-University Duesseldorf, Universitaetsstr. 1, 40225 Duesseldorf, Germany; 3 Core Facility Elektronenmikroskopie (CFEM), Heinrich-Heine-Universität Duesseldorf, Universitaetsstr. 1, 40225 Duesseldorf, Germany; 4 Department of Neurosurgery, University Hospital, Heinrich-Heine-University Duesseldorf, Moorenstr. 5, 40225 Duesseldorf, Germany

**Keywords:** Lacrimal gland, Microvascular anastomosis, Transplantation, Dry eye

## Abstract

**Introduction:**

Severe aqueous tear deficiency is caused by primary or secondary main lacrimal gland insufficiency. The transplantation of a human lacrimal gland could become a potential treatment option to provide physiological tears with optimal properties. To this end, we performed an ex vivo study to develop a surgical strategy that would ensure a vascular supply for a lacrimal gland transplant using microvascular techniques.

**Material and methods:**

Five cadaver heads were used to perform a lateral orbitotomy in order to identify the vascular pedicle and the lacrimal gland itself. The principal feasibility and the time of the required surgical steps for an intraorbital microvascular re-anastomosis of the human lacrimal gland were documented. Patency and potential leakage of the anastomosis were tested with hematoxylin intraoperatively. Postoperatively, routine histological, as well as scanning electron microscopy (SEM) of the gland and vascular anastomosis, were performed.

**Results:**

The vascular pedicle of all five glands could be isolated over a minimum stretch of at least 1 cm, severed, and successfully reanastmosed microsurgically. Time for arterial anatomization (*n* = 4) was 23 ± 7 min and 22 ± 3 min for the vein (*p* = 0.62). The total time for the entire microvascular anastomosis was 46 ± 9 min. All anastomosis were patent upon testing. SEM revealed well-aligned edges of the anastomosis with tight sutures in place.

**Conclusion:**

Our study demonstrates as proof of principle the feasibility of intraorbital microvascular re-anastomosis of a human lacrimal gland within the presumed window of ischemia of this tissue. This should encourage orbital surgeons to attempt lacrimal gland transplantation in humans in vivo.


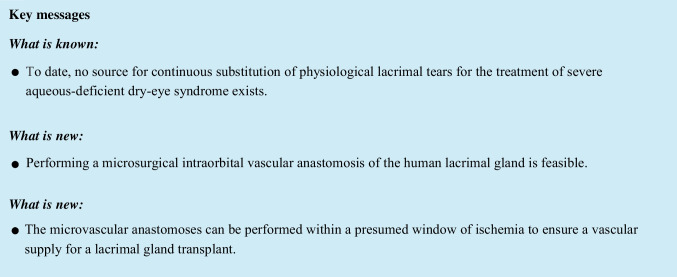


## Introduction

Severe aqueous tear deficiency is caused by primary or secondary insufficiency of the main lacrimal gland, e.g., in Sjoegren’s syndrome, Stevens-Johnson syndrome, or ocular cicatricial pemphigoid [1]. Affected patients often suffer from severe ocular surface irritation and pain and may require the application of tear substitutes every few minutes to relieve symptoms and prevent progressive ocular surface disease and loss of vision. The disease itself and the often insufficient therapy with lubricating eye drops or tear substitutes reduce the quality of life of the affected patients substantially [2]. Salivary gland transplantation, transposition of the parotid duct, or the use of mechanical dacryoreservoirs can improve Schirmer test result, break-up time and symptoms, and reduce the use of tear substitutes. While these are complex surgical procedures with potential for severe complications, none of them provides physiological tear and limits the success of these methods [3]. To date, no source for continuous substitution of physiological lacrimal tears for the treatment of severe aqueous-deficient dry-eye syndrome exists. While the transplantation of a human lacrimal gland could provide natural tears, this treatment option has neither been conceptualized before, nor any attempts have been made to establish a surgical technique to this end.

The functional and anatomic survival of a transplanted solid organ in general requires a vascular supply. Anastomosing the main lacrimal artery and vein would thus seem to be mandatory. During transplantation, the lacrimal gland would experience a period of interrupted arterial blood supply. This would have to be kept as short as possible since any tissue can tolerate only a certain time before ischemia results in extensive and irreversible damage. This time interval is termed “window of ischemia.” In practice, it is defined by the moment when the main blood-supplying artery is severed in the donor site and ends with the successful anastomosis of the artery to the vasculature of the recipient.

No data on the ischemia tolerance of the human lacrimal gland is available so far. For the submandibular and parotid gland, Sieg et al. showed in a rabbit model that after 90 min of ischemia, increasing structural damage to the secretory cells results, while no significant signs of tissue damage were observed with shorter periods of interrupted blood supply [4]. Since the lacrimal gland is—like the parotid gland—a predominantly serous gland, it is feasible to assume that an interruption of its blood supply for 90 min during transplantation will result in similar tissue damage and thus should be avoided. Therefore, a reliable, safe technique for successful and timely anastomosis of the lacrimal artery to the donor vasculature within a presumed window of 60 min is needed.

The aim of this study was to establish a surgical access as well as the principle microsurgical technique to identify and anastomose the human lacrimal gland vasculature in cadaver heads in order to assess the feasibility of microvascular lacrimal gland transplantation in humans that requires less than 90 min of surgical time.

## Methods

Five cadaver heads from the university hospital’s body donation program for medical education and scientific research were used to develop this surgical technique (Table 1). Three heads were preserved using the modified Thiel-soft-embalming protocol [5, 6]. This fixation procedure provides realistic visual and haptic properties without the risk of infection or time pressure due to tissue degradation. Briefly, fixation was achieved by initial perfusion and subsequent immersion in Thiel embalming fluid for at least 6 months. Solutions for veins, arteries, and each organ-system have their own composition of chemicals, containing different portions of water, p-chlorocresol, boric acid, ammonium nitrate, potassium nitrate, sodium sulfite, propylene glycol, ethylene glycol, formaldehyde solution, morpholine, and alcohol. The procedure was also performed in two unpreserved cadaver heads, which had been stored frozen overnight (12 h) before surgery. All procedures were performed by team of highly experienced ophthalmic and neurosurgical microsurgeons. In preparation for surgery, the heads were mounted on a regular ophthalmic surgery table. Standard ophthalmic surgical instruments, magnifying operating glasses for the lateral orbitotomy, as well as a surgical microscope (Leica M844 Type C40®) for the microvascular anastomosis were used.

**Table 1:** Gender, age, cause of death, type of fixation, and time from time-of-death (TOD) until surgery of the cadaver heads 1–5

Head	Gender	Age	Cause of death	Comorbidities	Fixation	Time (TOD to surgery)
1	Female	85	Sudden heart death	Cardiovascular disease, status post myocardial infarction, dementia	Thiel	Unknown
2	Female	74	Multi-organ failure	Acute respiratory distress syndrome, urosepsis, diabetes mellitus, arterial hypertension	Thiel	84 h
3	Male	87	Unknown	unknown	Thiel	50 h
4	Male	84	Multi-organ failure	Heart failure, pulmonary edema, peripheral artery occlusive disease	Frozen	38 h
5	Female	78	Cardiogenic shock	Cardiovascular disease, status post myocardial infarction	Frozen	19 h

A skin crease incision was performed with a D11 blade (Feather Disposable Scalpel®) in the lateral third of the upper eyelid crease extending over the lateral orbital rim. The subcutaneous soft tissues overlying the lateral orbital rim and the temporalis fossa were exposed bluntly using Steven’s scissors. The periosteum was incised and lifted off medially from the lateral orbital wall using a raspatory. The periosteum on the outside of the orbital rim was mobilized into the temporalis fossa before a routine lateral orbitotomy was performed. In brief, two horizontal orbitotomies, approximately 1.5 cm above and below the zygomaticofrontal suture, were performed using an oscillating sagittal saw (Medicon BienAir Microsagittal Saw®). Four holes, one above and one below each orbitotomy, were drilled using a 2 mm Rosen burr for later repositioning and fixation of the lateral orbital wall fragment at the end of surgery.

**Fig. 1:**
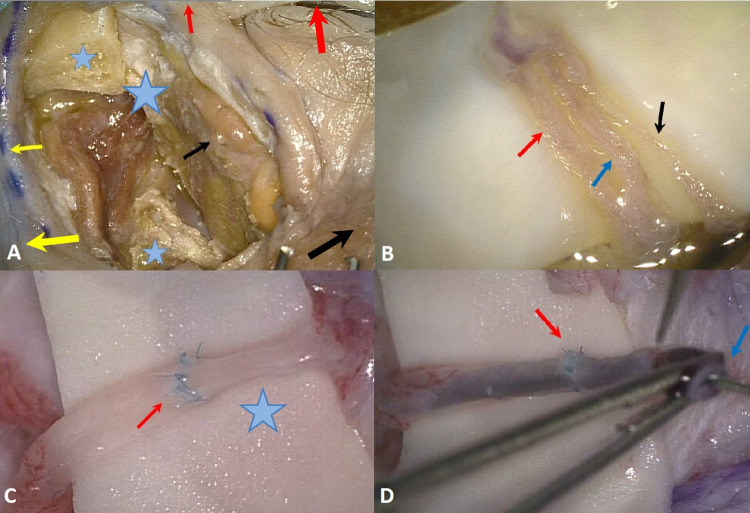
**A** Surgical field after lateral orbitotomy with visualization of the lacrimal gland (arrow): Blues stars mark edges of the orbitotomy (Orientation: inferior (red arrow), temporal (yellow arrow). **B** Lacrimal vasculature with artery (red arrow), vein (blue arrow), and nerve (black arrow) in head #1. **C** Complete anastomosis of the lacrimal vein (red arrow) in head #3. **D** Injection of hematoxylin (blue arrow) proximal to the anastomosis (red arrow) showing no signs of leakage or blockage of the vein in head #3

The cortical bone on the inside of the orbital wall was then thinned out using a burr before the lateral wall fragment was mobilized and temporarily removed. The periorbita overlying the lacrimal gland was opened using the D11 blade. The lacrimal gland was mobilized from the surrounding orbital fat. The vascular pedicle, which lies at the posterior surface of the gland, was identified (Fig. 1A). A latex sheath was placed under the vascular pedicle, and the individual vessels of the lacrimal vasculature were carefully isolated (Fig. 1B). Care was taken to clean the outer vessel wall from all excess fibrous tissues, but to leave the adventitia intact. Subsequently, the artery and vein were severed using vannas scissors (Geuder AG, Heidelberg, Germany). Next, a vascular microanastomosis of both vessels was performed end to end using 10.0 Prolene interrupted sutures (Fig. 1C). Depending on the vessel size, four to six single-knot sutures were placed. The vessels were then again severed distant to the anastomosis and patency check was performed with a hematoxylin solution injected with a blunt cannula into the lumen to evaluate for leakage or blockage (Fig. 1D).

Finally, the lacrimal gland with the re-anastomosed vasculature was explanted. The lateral bony orbital wall fragment was repositioned and fixed with 4.0 Vicryl interrupted sutures using the predrilled holes. The subcuticular tissues and skin were closed layer by layer with 6.0 Vicryl-interrupted sutures. All procedures were videotaped and the time required for the microvascular steps was analyzed. At the end of the procedure, the surgeons evaluated the haptic properties of the soft tissue on a scale from 0 to 3 (0 = completely disintegrated, 1 = gland + vasculature identified without the ability to anastomose, 2 = gland + vasculature identified with the ability to anastomose, but inferior to in vivo situation, 3 = equivalent to in vivo situation).

The explanted glands, including their vascular pedicle, were postfixated in 4% buffered formaldehyde, dehydrated with Leica® TP1020 automatic tissue processor, and vacuum paraffin-wax embedded using the Medite® TES Valida embedding center. 5 µm slices of the tissue were cut using a microtome Leica® RM2155 before the sections were stained with hematoxylin and counterstaining eosin for qualitative assessment. Measurement of the lumen size was taken based on the hematoxylin/eosin slide using the software ImageJ (NIH, with Java 1.8). Scanning electron microscopy was used to visualize the anastomosis. The fixated samples were dehydrated using acetone (50%, 70%, 90%, and absolute) and subsequently critical point dried using a Leica EM CPD030 (Leica Biosystems, Wetzlar, Germany). A thin layer of chromium was sputtered onto the sample (Q150T ES, Quorum Technologies, Laughton, East Sussex, UK). SEM images were obtained using a Zeiss Crossbeam 550 (Carl Zeiss Microscopy, München, Germany) at 3 kV.

## Results

In all five heads, the surgical steps including opening the periorbita could be performed. In four heads the lacrimal gland and its vasculature could be identified and soft tissue quality was deemed suitable for the microanastomosis. In head #2, the intraorbital soft tissues, including the lacrimal gland and its vasculature, were found to be too badly preserved. This head had also been stored for the longest period in the Thiel fixation solution (Table 1). In the remaining 4 heads (2 Thiel fixated and 2 non-fixated/ “fresh”) the tissue quality was sufficient to perform the microsurgical steps, but the texture of the intraorbital tissues varied depending on the type of fixation (Table 2). Most realistic haptic properties were found in a non-fixated, non-frozen cadaver head with a short period between death and surgery.

**Table 2:** Success of surgery, time needed for anastomoses, surgeon, and evaluation of haptic properties of the cadaver heads 1–5

Head	Anastomoses	Time for arterial anastomoses (min.)	Total time for anastomoses (min.)	Evaluation of haptic properties
1	Successful	15	38	3
2	Not attempted	-	-	0
3	Successful	33	57	2
4	Successful	25	48	3
5	Successful	22	39	3

**Fig. 2:**
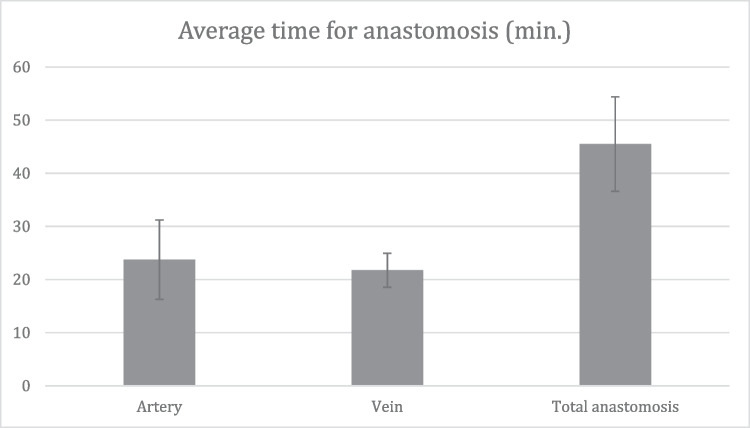
Average time for performing an anastomosis of the artery and vein (*n* = 4)

**Fig. 3:**
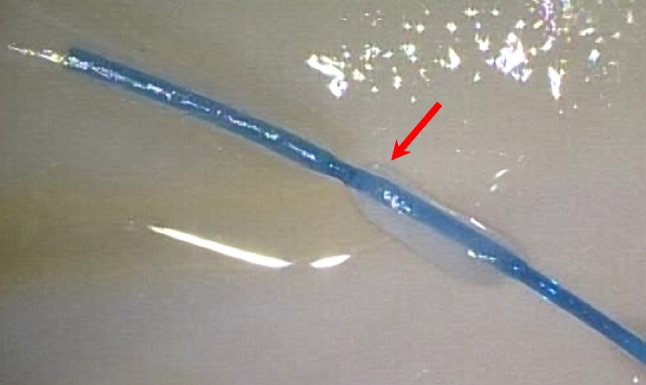
4.0 Prolene suture as a bridge to keep both lumen at the end of the anastomosis (red arrow) open in head #3

**Fig. 4:**
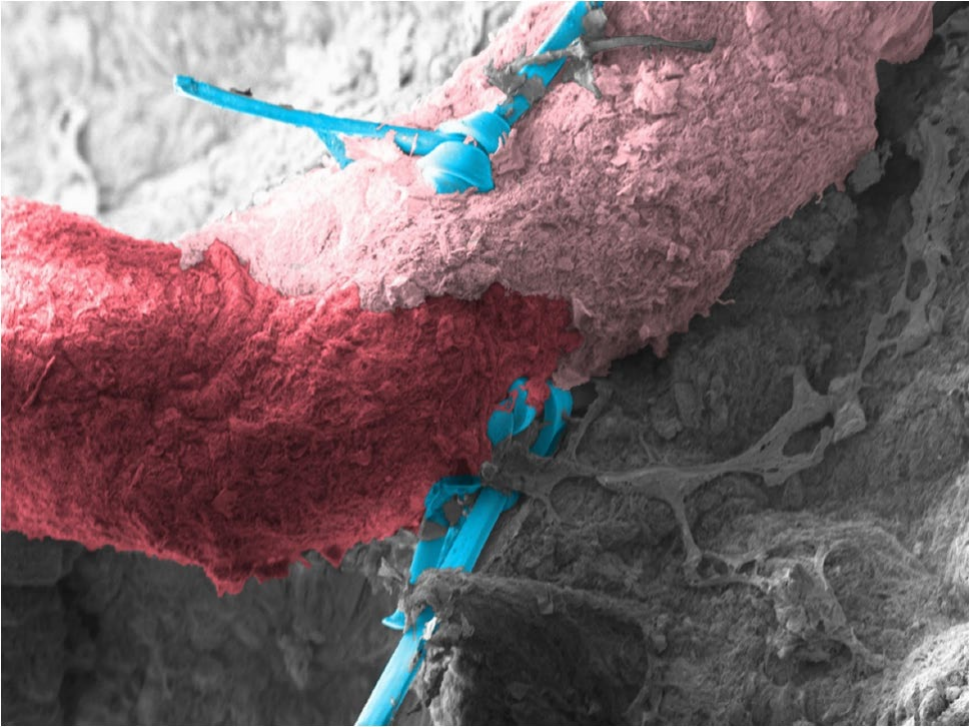
Colored SEM image with well-aligned/anastomosed ends of the artery (red donor and pink recipient) as well as the prolene suture (blue) in place (head 3)

**Fig. 5:**
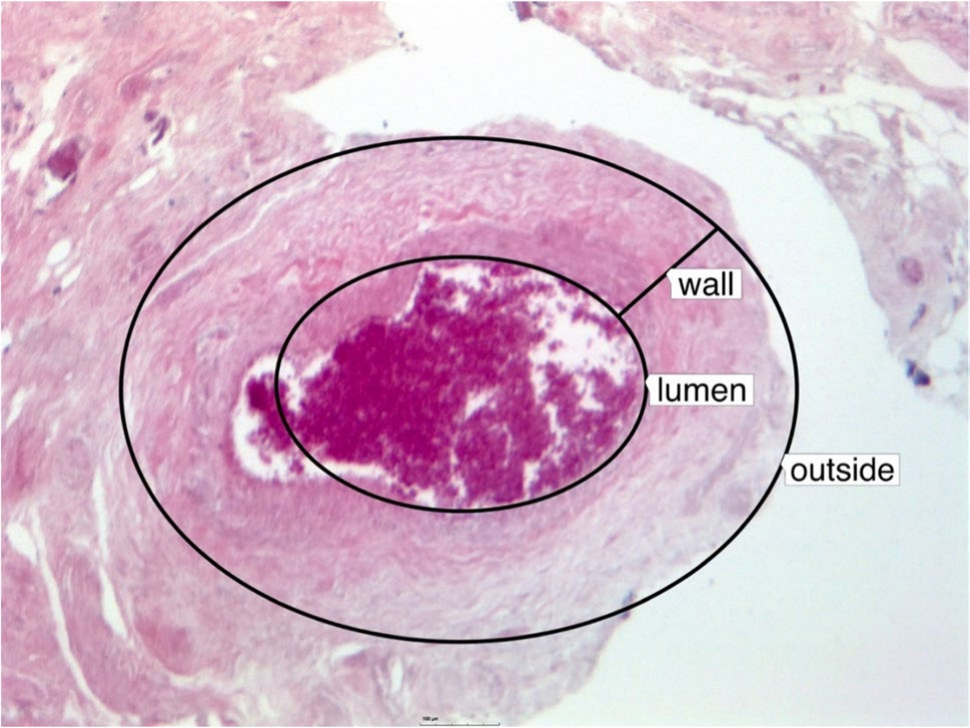
Histology of the arterial vessel showing the size of the lumen compared to its wall. Lumen: min–max diameter: 160–234 µm, wall: min–max diameter 317–425 µm (head #1)

In four heads a stretch of the vascular pedicle of at least 1 cm could be identified and all microsurgical steps performed. The surgical time for the microanastomosis (*n* = 4) of the lacrimal artery was 23 ± 7 min and 22 ± 3 min for the vein. The total time for the entire microvascular anastomosis was 46 ± 9 min. (Fig. 2). There was no statistically significant difference between the time to perform the arterial or the venous anastomosis (*p* = 0.62). There was also no statistically significant difference in the surgical time between the frozen or the Thiel fixated cadavers, neither for the arterial (*p* = 0.48) nor for the venous anastomoses (*p* = 0.41). In one case, the lumen of the vein collapsed on both ends of the anastomosis so that a suture with 4.0 Prolene was used as a temporary bridge to hold the lumen open (Fig. 3). Check with hematoxylin revealed no signs of blockage or leakage from all anastomoses (Fig. 1D). SEM revealed well-aligned edges of the anastomosis with tight sutures in place (Fig. 4). Histology of the tissue revealed the characteristic morphology of the human lacrimal gland. The diameter of the lacrimal artery (head #1) was measured to be approximately 320 microns (Fig. 5).

## Discussion

Surgical treatment options to substitute lubrication in severe aqueous tear deficiency are so far limited to the transplantation of salivary glands or the use of mechanical pump dacryoreservoirs [7, 8]. Both, transplanted salivary glands and subcutaneous abdominal pump reservoirs with catheter connection to the upper conjunctival fornix have been advocated for patients with the most severe form of dry eye only and do not provide true, i.e., physiological tears. Transplantation of a human lacrimal gland could potentially restore a completely natural tear secretion. Successful human lacrimal gland transplantation would require (A) a safe surgical technique, (B) adequate perioperative logistics, and (C) specific postoperative treatment to ensure long-term viability and function of the gland.

### Surgical technique

Harvesting the gland from the donor site, be it allogeneic (or less likely autologous), will invariably require a lateral orbitotomy to identify the vascular pedicle of the lacrimal gland. Depending on the patient’s anatomy, the osteotomy described here may pose the risk of intracranial or frontal sinus extension [9]. While a skilled orbital surgeon should be able to avoid such complications, it seems justified to use a multidisciplinary surgical team of ophthalmologist and neurosurgeon for such a complex procedure.

The viability of a transplanted whole, solid organs such as the kidney requires a functional blood supply [3, 5, 10]. In some organs, such as the pancreas, a total ischemia time of over 12 h is acceptable to provide good functionality, but in salivary glands, Sieg et al. found that this is limited to a maximum of 90 min, before irreversible tissue damage results in permanent loss of functionality [4, 11]. Arterial blood supply to these grafts thus has to be restored within this window of time. In the absence of any data on the ischemia tolerance of the lacrimal gland, it is feasible to assume that its arterial blood supply should during transplantation also be restored in less than 90 min to avoid permanent tissue damage. Hence, after harvesting the graft from the donor a rapid microvascular arterial anastomosis in the recipient would be a vital step for success.

A surgical technique for microvascular anastomosis of excretory glands such as the submandibular gland has already been established. For the lacrimal gland—probably due to the complex anatomical situation—this has never been attempted so far. The lacrimal vessels have a hidden intraorbital position entering the lacrimal gland on its back surface, making surgical access difficult. Here, we first describe a surgical technique via a lateral orbitotomy approach that can be used to successfully identify and reanastomose the lacrimal artery and vein. In our series adequate isolation of the lacrimal gland and its vascular pedicle, consisting of one or two arteries and one vein, was possible in all cases if tissue preservation was sufficient. The dimensions of the lacrimal vessels make microscopy assistance a prerequisite for more delicate surgical maneuvers. Via the approach described here the vascular pedicle could be isolated and reanastomosed in all specimens. The all-important arterial anastomosis was completed within on average 23 min and in a maximum of 33 min, thus well respecting the presumed window of ischemia. All anastomosis proved to be patent and thus functional.

In vivo visualization of microstructures such as vessels may be more difficult due to bleeding, tissue edema, or atrophy of the recipient’s vessels, e.g., secondary to chronic lacrimal gland destruction. These problems were all absent in our cadaver model but could prolong the time needed to perform essential surgical steps, such as the micro-anastomosis, in vivo. However, the longest total time for the two anastomoses in one case was 57 min. With an expected window of ischemia of 90 min to complete the arterial anastomosis alone, there is a sufficient time margin even in case of surgical delay [4]. Furthermore, the arterial anastomosis would always be performed first and once achieved, flushed, e.g., with heparinized saline to prevent micro-clots, and then intermittently be opened to reoxygenate the graft. Alternatively, other vascular options in the recipient could more easily be accessed, e.g., the supraorbital neurovascular bundle or branches of the temporal artery, but this requires additional study. Further studies will also need to establish the functionality of and exclude leakage from the vascular anastomosis in vivo.

The lacrimal gland is part of a reflex effector system responding to triggers of corneal sensation. This reflex tearing is also dependent on an intact innervation of the lacrimal gland via the ophthalmic branch of the trigeminal nerve [12]. In other solid organs transplants such as the kidney evidence of reinnervation into the graft was observed as early as 5 months postoperatively without any surgical nerve anastomosis, probably originating from recipient ganglions located near the arterial anastomosis [13]. Thus, the microvascular anastomosis could also serve as guidance for nerves regrowing into the lacrimal gland graft. In addition, we found in biopsies of submandibular glands after free autotransplantation that parasympathetic ganglion cells survive in the grafts for many years and provide an autonomous source of neural stimulation to the secretory acinar cells [14]. From free submandibular gland transplants, we have learned that the grafts remain viable despite complete separation from their normal nerve supply and show an increasing secretory activity over months with excellent long-term functionality [15]. Thus, a nerve anastomosis or alternatively an additional neurotization procedure may also not be needed in lacrimal gland transplantation [16].

Challenges not addressed in our study are the steps of harvesting and implanting the entire lacrimal gland with attached secretory ductules and conjunctiva as well as fixation of the donor organ in the recipient’s orbit. However, transplanting a complex of lacrimal glands with ductules and conjunctiva should be no major challenge for a trained orbital surgeon since a lateral orbitotomy approach allows good access and visualization of the superotemporal fornix. Ultimately, this should offer the additional benefit to address conjunctival scarring often present in the cohort of patients likely to benefit from lacrimal gland transplantation, such as individuals with severe Graft versus Host Disease, Stevens Johnson-syndrome or cicatricial mucous membrane pemphigoid [3].

### Perioperative logistics

Following donor and recipient informed consent, the transplantation would likely be performed on short notice. The organ harvest would have to be performed while the donor’s blood circulation is maintained. Hence, only multiorgan donors (not including autologous transfer, e.g., from left to right) would be suitable, and donor organ retrieval would have to be coordinated with other surgical specialties, such as cardiothoracic surgeons, removing other organs [17]. The surgical team would initially benefit from a collaboration of an orbital surgeon with, e.g., neuro- or maxillofacial surgeons with expertise in microvascular anastomosis, but in principle, a well-trained orbital surgeon should be in the position to perform lacrimal transplantation on his own. To minimize the window of ischemia, two teams would be required to perform surgery simultaneously, more or less side by side, having duplicate surgical equipment at hand [18]. While one team would have to retrieve the graft, a second team would have to prepare the recipient site and in particular the vascular pedicle.

### Postoperative treatment

In the postoperative period, a short treatment phase of systemic antibiotics and anticoagulants should be considered. The most likely critical problems are vascular complications (e.g., dehiscence or obstruction, e.g., by thrombosis, of the vessels) and—rarely—the obstruction of secretory ducts. In submandibular gland transplantation performed for severely dry eyes by experienced surgical hands, such complications were rare, i.e., below 10%. In our own cohort, we reported a viability of these grafts of 80% during a follow-up of over 5 years [15].

For allografts, postoperative management will have to include long-term systemic immunosuppression of the recipient [19–21]. Hence, human lacrimal gland allotransplantation will only be justified in patients with the most severely dry eye and potentially require HLA-matching. However, in such a cohort ocular disease often is a result of severe systemic inflammatory conditions and associated blindness requires high-risk corneal grafting. The potential recipients of lacrimal gland grafts, therefore, are likely to either already receive or—if initiated—benefit from disease-modifying immunosuppression and long-term general medical screening [15].

### Perspective

Techniques for extended organ survival after harvest could potentially be transferred from other applications to lacrimal gland transplantation in order to expand the window of ischemia. This could, e.g., include hypothermic storage of the donor organ. In kidney transplantation, temperatures as low as 4 °C are used to reduce tissue metabolism after organ retrieval by 90 to 95% and thus, decrease the consumption of ATP [10]. Storage solutions and perfusion devices, which are used for donor kidneys, also reduce vascular resistance and thus increase perfusion during storage [22].

In addition, some of the surgical steps described here could potentially be useful for other therapeutic approaches, such as the implantation of engineered secretory active, three-dimensional constructs [11, 23–26]. While many obstacles will have to be overcome before lacrimal gland transplantation can be inaugurated in the living human, our study demonstrates the general feasibility of performing a microsurgical intraorbital vascular anastomosis of blood vessels within the presumed window of ischemia. It thus lays the foundation for further research in preparation for human lacrimal gland transplantation or implantation of engineered vascularized tissue constructs.

## References

[1] Nebbioso M, Del Regno P, Gharbiya M, et al (2017) Analysis of the pathogenic factors and management of dry eye in ocular surface disorders. Int J Mol Sci 18. 10.3390/ijms18081764 10.3390/ijms18081764 PMC5578153 28805710

[2] Barabino S, Labetoulle M, Rolando M, Messmer EM (2016). Understanding symptoms and quality of life in patients with dry eye syndrome. Ocul Surf.

[3] Geerling G, Sieg P, Bastian GO, Laqua H (1998). Transplantation of the autologous submandibular gland for most severe cases of keratoconjunctivitis sicca. Ophthalmol.

[4] Sieg P, Geerling G, Kosmehl H (2000). Microvascular submandibular gland transfer for severe cases of keratoconjunctivitis sicca. Plast Reconstr Surg.

[5] Kluckman M, Fan J, Balsiger H (2015). Clinical considerations of the glandular branch of the lacrimal artery. Clin Anat.

[6] Wolff K-D, Kesting M, Mücke T (2008). Thiel embalming technique: a valuable method for microvascular exercise and teaching of flap raising. Microsurg.

[7] Geerling G, Brewitt H (2008) Surgical management of dry eye part II. In: Geerling G, Brewitt H (eds) Surgery for the Dry Eye. Karger 326

[8] Murube J, Geerling G (2008). Mechanical pump dacryoreservoirs. Dev Ophthalmol.

[9] Alzhrani GA, Gozal YM, Sherrod BA, Couldwell WT (2019). A modified lateral orbitotomy approach to the superior orbital fissure: a video case report and review of anatomy. Oper Neurosurg (Hagerstown, Md).

[10] Requião-Moura LR, de Durão JMS, de Matos ACC, Pacheco-Silva A (2015). Ischemia and reperfusion injury in renal transplantation: hemodynamic and immunological paradigms. Einstein (Sao Paulo).

[11] Wakamatsu TH, SantʼAnna AEBPP, Cristovam PC (2017). Minor salivary gland transplantation for severe dry eyes. Cornea.

[12] Dua HS, Said DG, Messmer EM (2018). Neurotrophic keratopathy. Prog Retin Eye Res.

[13] Tantisattamo E, Molnar MZ, Ho BT (2020). Approach and management of hypertension after kidney transplantation. Front Med.

[14] Singh S, Basu S, Geerling G (2022). Salivary gland transplantation for dry eye disease: indications, techniques, and outcomes. Ocul Surf.

[15] Borrelli M, Schröder C, Dart JKG (2010). Long-term follow-up after submandibular gland transplantation in severe dry eyes secondary to cicatrizing conjunctivitis. Am J Ophthalmol.

[16] Lueke JN, Holtmann C, Beseoglu K, Geerling G (2020). Corneal neurotization. Ophthalmologe.

[17] Casanova D, Castillo F, Miñambres E (2022). Multiorgan retrieval and preservation of the thoracic and abdominal organs in Maastricht III donors. World J Transplant.

[18] Casanova D, Gutierrez G, Gonzalez Noriega M, Castillo F (2020). Complications during multiorgan retrieval and pancreas preservation. World J Transplant.

[19] Yu K, Lian X-F, Jiang X-Y, Zhou S-Y (2021). Efficacy of immunosuppressants in high rejection risk keratoplasty: a meta-analysis of comparative studies. Cornea.

[20] Abudou M, Wu T, Evans JR, Chen X (2015). Immunosuppressants for the prophylaxis of corneal graft rejection after penetrating keratoplasty. Cochrane Database Syst Rev.

[21] Yaïci R, Roth M, Juergens L, et al (2022) On the current care situation and treatment of ocular mucous membrane pemphigoid in Germany. Klin Monbl Augenheilkd. 10.1055/a-1720-1819 10.1055/a-1720-1819 35609814

[22] Lee CY, Mangino MJ (2009). Preservation methods for kidney and liver. Organogenesis.

[23] Spaniol K, Metzger M, Roth M (2015). Engineering of a secretory active three-dimensional lacrimal gland construct on the basis of decellularized lacrimal gland tissue. Tissue Eng Part A.

[24] Dietrich J, Schlegel C, Roth M (2018). Comparative analysis on the dynamic of lacrimal gland damage and regeneration after Interleukin-1α or duct ligation induced dry eye disease in mice. Exp Eye Res.

[25] Massie I, Spaniol K, Barbian A (2018). Development of lacrimal gland spheroids for lacrimal gland tissue regeneration. J Tissue Eng Regen Med.

[26] Massie I, Dietrich J, Roth M (2016). Development of causative treatment strategies for lacrimal gland insufficiency by tissue engineering and cell therapy. Part 2: reconstruction of lacrimal gland tissue: what has been achieved so far and what are the remaining challenges?. Curr Eye Res.

